# Community-based social healthcare practices in China for healthy aging: a social prescription perspective analysis

**DOI:** 10.3389/fpubh.2023.1252157

**Published:** 2023-10-02

**Authors:** Rashid Menhas, Lili Yang, Rana Danish Nisar

**Affiliations:** ^1^Department of Nursing, The Fourth Affiliated Hospital, Zhejiang University School of Medicine, Yiwu, Zhejiang, China; ^2^Department of Politics and International Relations (DPIR), University of Sargodha, Sargodha, Pakistan

**Keywords:** community, social healthcare, practices, social prescription, nature, culture, healthy living

## Abstract

**Background:**

The global population is aging, and the number of people suffering from chronic diseases is increasing. In response to these trends, community-enhanced social healthcare practices are a novel paradigm of social prescribing that aims to improve both the community's and the individual's level of health by combining community involvement, organizational change, and individual-level practice.

**Objective:**

The study examined the state of community-based social healthcare practices using the lens of social prescription in China with an eye on promoting healthy aging there.

**Method:**

Thematic analysis approach was used in this investigation. A social prescription lens was used to conduct an open-ended theme study of China's community-based social healthcare practices for healthy aging. The research was conducted in Yiwu in Zhejiang Province, P. R. China. A sample of 24 “comprehensive evaluation team members (CETM)” was chosen using a purposive selection strategy.

**Results:**

In the context of the social prescription, we analyzed social healthcare practices for healthy aging at the community level. All the comprehensive evaluation team members described community social healthcare practices under the paradigm of social prescription. After analyzing the community social healthcare practices under the paradigm of social prescription, six main themes (E-Social Prescription, Nature-based Social Prescription, Healthy Living Social Prescription, Culture-based Social Prescription, Health Screening Social Prescription, and Health Education Social Prescription) emerged for healthy aging at the community level.

**Conclusion:**

Social prescribing links individuals to non-clinical services and activities, typically provided by the nonprofit and community sectors. Community-based social healthcare practices under social prescription can be an efficient and cost-effective way to assist patients with chronic diseases in managing their illnesses and enhancing their overall health and wellbeing.

## Introduction

There will be significant challenges for China's healthcare system as the country's population ages. By 2050, China will have a population of 1.4 billion, with 400 million people aged 65 and over and another 150 million aged 80 and up. There is already a lot of pressure on the modern public healthcare system, and it's only worsening as the number of older adults cared for by their traditional families decreases (1). The Chinese government has taken several preventative measures toward a long and healthy life expectancy, including the “Healthy China 2030” planning blueprint (2). The Chinese government established a national medical insurance policy to address the rising medical costs of middle-aged and older adult citizens (3). The “Healthy China Action” program, which runs from 2019 to 2030, promotes healthy aging and aims to improve the health of the Chinese people as a whole (4). Promoting healthy lifestyles, bolstering social welfare, and bettering the environment are all examples of non-clinical acts included in the plan (5). The Chinese government promotes healthy aging by integrating nursing and healthcare for older adults (6). The significance of community-based approaches to social healthcare in promoting healthy aging in China is growing in importance. Community pharmacies often serve as patients' first entry points into the healthcare system. They have provided many public health services, but those dealing with the prevention and treatment of drug abuse, the promotion of a healthy diet and lifestyle, the reduction of risk factors for infectious illness, the improvement of prevention for cardiovascular disease, and the aiding in the cessation of smoking have received the most attention (7). Community healthcare for seniors has been researched, as have the elements that affect seniors' preferences regarding their older adult care (8).

China promotes healthy aging via community-based social healthcare. It includes community occupational therapy, appropriate eating programs, social interaction strategies, and exercise (9). Public health therapists provide a broad range of services, including help with quitting smoking, improving one's diet and way of life, and warding against or treating conditions including cardiovascular disease, infections, drug abuse, and addiction (1, 2). Engaging in social activities is beneficial for the healthy aging of Chinese seniors. To age well, one needs social support, stable housing, and active societal membership. Objective social support includes having a safe place to live and accessing necessary resources; subjective social support includes experiencing encouragement and acceptance. For older adults, objective social assistance is more effective than subjective help (10). The cognitive abilities of Chinese seniors are boosted by their participation in communal activities (11). Participation in social activities, financial stability, the presence or absence of chronic health conditions, and the ability to provide for grandchildren are among the most important factors in the health of older adult Chinese (12). Chinese seniors who don't actively participate in their communities are less likely to age healthily. Active social lives are associated with better cognitive performance, episodic memory, and visual-spatial ability among Chinese seniors (13). Volunteering, joining a social club, and going to church are all examples of what we mean by “social participation.” Social support has influenced the relationships between social engagement, wellbeing, and mental health issues among Chinese seniors. Their social interactions have also greatly impacted seniors' mental health (14). Physical health and suicidal thoughts in rural Chinese seniors correlate, although social engagement mediates this relationship (15). Social determinants of health screening are important for good aging among Chinese seniors, and traditional attitudes such as pessimism, self-overhaul, and the sizzling and excellent balance have an effect. The perspectives of older adult Chinese women on fitness, ailment, and anticipatory medicine are influenced by these notions. Ethnic sights, dialectal blockades, doctor-patient communiqué, and entrée to health care are all potential cancer risk factors in this population (16). Since isolation is a major and independent risk factor for depression in older adults, (17, 18) it is beneficial for the subjective wellbeing of older Chinese people to get support from their offspring. Community healthcare practices are important to the social prescription for good aging. These practices involve co-creating individualized strategies to promote patients' health and wellbeing in the voluntary, community, and social enterprise sectors (19). Social medicine aims to enhance patients' quality of life by reducing their feelings of isolation and anxiety and assisting patients experiencing difficulties with their homes or finances (20). Community development is important to social medicine because it can strengthen a community's cohesiveness and improve individuals' health (21).

### Social healthcare practices for health and wellbeing at community level

Participation in community-based programming that encourages healthy aging has been linked to higher levels of social engagement among older adults (22). According to studies (23, 24), elders who are socially engaged and invested in their communities can better keep their health in check and live independently as they age. Participation in communal activities, such as joining a club or society, is associated with a lower risk of dementia (25). Participating in social activities and maintaining relationships with one's family and friends are important for healthy aging. Studies have indicated that maintaining social connections and engaging in socially beneficial activities, such as continuing to work, gaining new skills, attending cultural events, and volunteering, can help people maintain their health as they age (26). Screening for various health conditions within the community is essential to assist people in aging healthily. It is critical to provide systematic screening as a high priority for high-risk groups and to follow up screening with more accurate evaluations and the appropriate therapies (27).

Additionally, it is essential to prioritize systematic screening for low-risk groups. Community health care providers can also use alterations in health status as a screening criterion to encourage health screening (28). Education about health is one of the most important aspects of helping older people mature healthily. It has been demonstrated that community-based health education interventions are beneficial in preventing frailty in older persons, encouraging physical exercise, nutrition, and social involvement, optimizing nutritional status, and lowering depression, anxiety, and stress (29–33). People should be taught through health education programs how to take better care of themselves, how to live and behave healthily, and how to obtain knowledge and resources related to health to maintain the excellent health of the older adult members of the community (32). It has been demonstrated that approaches to public health, such as community interventions to prevent frailty, can assist older individuals in growing older in a healthy manner (33). The general public needs to be aware of how vital health screenings are for senior citizens, as well as for senior citizens themselves, to be aware of how important it is to maintain their health. Furthermore, it is essential for families and employees working in community health centers to be aware of how to monitor the health of senior citizens. Getting a nutritional assessment, becoming a member of a club in the community that caters to seniors, and receiving instruction on how to exercise precisely are three of the most typical components of health promotion programs for older citizens (34).

### Social prescription for health and wellbeing in a community setting

Social prescription, also known as community referral or social prescribing, is an all-encompassing approach to improving community members' health and wellbeing. Exercise groups, art therapy, and gardening clubs are examples of non-medical support services and community-driven activities linked via this method (35). Supplementary, folks are turning to societal prescriptions to improve their health and the health of their communities. Understanding that health and wellbeing are affected by a wide range of circumstances outside those within the purview of conventional medicine is the basis of social prescribing. Social prescription promotes community health and wellbeing by focusing on underlying social factors. A social prescription may promote mental and physical health, increase social support, and decrease dependency on healthcare services by linking people with non-medical support services and activities customized to their requirements (36). Nursing practitioners' potential interactions with social prescribing platforms to strengthen communities' resilience are outlined in social prescription, a subset of the broader NHS England concept for universal customized care. During home visits or trips to community clinics, community nurses evaluate their patient's psychological and social wellbeing. They help determine who needs help beyond medical care and might benefit from a social prescription approach. Community nurses assess their patient's needs and interests before directing them to the most suitable community-based activities and services (such as support groups, fitness courses, arts and crafts workshops, and volunteer opportunities). Community nurses collaborate with other healthcare team members to create individualized patient treatment plans. Patients are followed up with by community nurses so that the efficacy of the social prescription method may be assessed. To ensure the treatment plan is still useful and up-to-date, they provide input to patients, their families, and other medical staff. Patients and their loved ones may learn much from community nurses about living healthier and more active lives just by listening to what these professionals say. To offer social prescription services and guarantee that patients have access to various resources and assistance, community nurses build and maintain partnerships with local organizations, service providers, and community groups (37). Practices of social prescribing move medical care outside of traditional professional settings and focus on how social circumstances influence an individual's health (38). Healthy aging rounds consist of one-on-one conversations with mentors who are healthy older individuals. During these conversations, participants practice assessment, interviewing, and counseling for social support and physical activity (39).

### The current study

**Figure 1:**
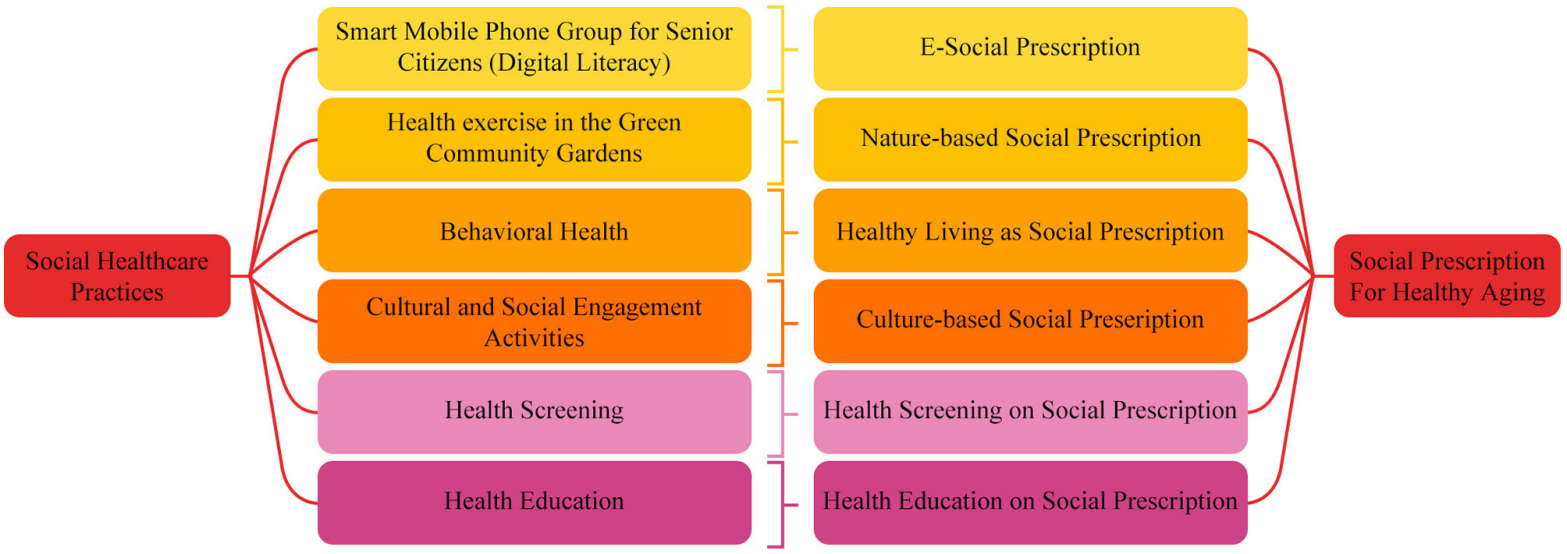
Conceptual framework.

Older people's participation in community health screenings, which has become a hot subject in China, has been discovered to be influenced by factors such as self-rated health, physical condition, and mental health (40). However, there is a lack of research comparing and contrasting different strategies. So, it's important to evaluate the impact of various social healthcare programs and variables on healthcare services and expenditures incurred by the Chinese middle-aged and older adult (41). It has been suggested that big data and machine learning may be used to identify the factors impacting older adults' health conditions and life satisfaction (42). Integrating a strong legal framework with such services is also being studied (43) to encourage the healthy and sustainable growth of older adult care services. It has recently come to light in China that community-based social healthcare projects may effectively foster good aging. Non-medical social prescription has gained popularity as a means of addressing social determinants of health by linking individuals to community resources that may improve their health and wellbeing. China has launched a community-led social prescribing effort with the help of 40 local organizations. The majority of research and attention has gone into improving the mental health and supplying the psychosocial needs of seniors via local organizations. Primary care community health facilities were used in the original project's integration into the larger health system infrastructure to screen for health-related social needs for seniors during their yearly check-up. The function of the link worker was taken on by community health professionals, social workers, and the larger mental health support team, who worked with each individual to match them with the best community resources for their specific situation. For this reason, the “comprehensive evaluation team” was used in place of the term “link workers” (44) to reflect the fact that it was not just one profession that was responsible for bridging the gap between medical and social care. Since then, recommendations for expanding social prescribing have been published (45), and the Chinese Society of Geriatric Psychiatry has supported a proposal to expand the program. Although non-clinical community-based social healthcare practices in China effectively promote healthy aging, there is a shortage of information on their applicability and effectiveness, especially from a social prescription perspective. Using the lens of social prescription (see Figure 1), this study will examine the state of community-based social healthcare in China with an eye on promoting healthy aging there. This study will also help enhance the quality of life for older adults by shedding light on the efficacy of community-based social healthcare practices from the standpoint of social prescription.

## Methodology

### Study design

Thematic analysis, developed by Braun and Clarke (46), was used in this investigation. A social prescription lens was used to conduct an open-ended theme study of China's community-based social healthcare practices for healthy aging. The research was conducted in Yiwu in Zhejiang Province, China.

### Study participants

It is common practice in qualitative studies to choose samples for certain reasons. Researchers contacted the “comprehensive evaluation team” through text messages and wechat. In China, 40 community partners have participated in a grassroots campaign to practice social prescription. Older folks' mental health and psychosocial needs have been the main focus of community programs. The concept was initially included in the more significant infrastructure of the health system by using primary care health centers in the community to detect health-related social needs during older individuals' yearly health examinations. A group of health professionals in the community, social workers, and other mental health care specialists took on the job of a link worker. By referring to this group as the “comprehensive evaluation team” rather than just “link workers,” it recognizes the multifaceted skills of the healthcare workers who fill the divide between social and medical services (47). The Chinese Society of Geriatric Psychiatry has given their support for the desire to increase the scope of this project, and they have also released recommendations for implementing social prescribing on a bigger scale. A training program for the team doing the comprehensive review has also been created through collaboration. With the help of this curriculum, community services are adequately referred to, and effective screening for psychosocial needs is ensured. As part of the Healthy China 2030 Action Plan, China is moving its national strategy toward illness prevention and health maintenance. Although social prescription benefits society and the health system beyond merely older people, it is nevertheless vital to promote it (48). A sample of 24 “comprehensive evaluation team members (CETM)” was chosen (see Table 1) using a purposive selection strategy.

**Table 1:** Demographics of the participants.

**Participants**	**Age (years)**	**Education**	**Gender**	**Working experience (Years)**	**Work station name**
CETM 1	29	Master	M	4	Jiangbin Community
CETM 2	30	Graduate	F	5	Shengli Community
CETM 3	27	Bachelor	F	3	Linjiang Community
CETM 4	28	Bachelor	F	3	Jimingshan Community
CETM 5	31	Master	F	5	Dongzhou Community
CETM 6	32	Bachelor	M	3	Taxiazhou Community
CETM 7	26	Graduate	F	3	Wanghu Community
CETM 8	29	Master	M	4	Tonghui Community
CETM 9	28	Master	F	3	Xiuhu Community
CETM 10	33	Master	M	3	Wuai Community
CETM 11	30	Graduate	F	4	Xuefeng Community
CETM 12	28	Master	M	4	Jiangnan Community
CETM 13	35	Master	M	6	Yinyuan Community
CETM 14	27	Master	M	3	Xiaozi Temple Community
CETM 15	34	Graduate	F	5	Fenghuang Community
CETM 16	25	Bachelor	F	3	Jindu Community
CETM 17	29	Master	F	4	Liming Community
CETM 18	31	Graduate	F	5	Xiangxi Community
CETM 19	26	Master	M	4	Songmenli Community
CETM 20	37	Graduate	M	6	Shangbo Community
CETM 21	36	Graduate	F	5	Panxi Community
CETM 22	39	Graduate	F	7	Binwang Community
CETM 23	38	Graduate	F	7	Liuci Community
CETM 24	37	Graduate	M	8	Jianshe Community

CETM, Comprehensive evaluation team member.

### The procedure

#### Data collection instrument

Data for this investigation were gathered with the use of an interview guide. The interview guide employed open-ended questions that allowed participants to elaborate on the study's major research question. Social healthcare practices within the social prescription paradigm were the focus of the inquiries, as were the methods for classifying such social healthcare activities. With Patton's approach, this interview guide was written (49). In this type of interview, the interviewers who follow a strict script strictly follow the question language and order. The interviews are still considered qualitative rather than quantitative because the responses are open-ended. It is the most systematic and efficient qualitative interviewing technique and helps reduce biases.

### Data collection

Interviews with community people who were part of the “comprehensive evaluation team” were conducted by researchers using a purposive sampling strategy. To make the most of limited resources, qualitative researchers often use the purposeful sampling approach (49). It involves selecting examples that are statistically more likely to contain useful information about the topic at hand. Each research participant was interviewed both in-person and remotely, using a hybrid approach that considered their schedule and preferences. Participant permission was gained prior to their interview once the study's goal was explained.

### Data analysis

#### Quality check of the interview data

Members checked each other's work to ensure the accuracy of the data obtained during interviews. One method for ensuring the reliability of survey findings is via member checks, also known as participant or respondent validation. Data or outcomes are sent back to participants to double-check the accuracy and consistency of their accounts (50).

#### Final data analysis

The interview data were subjected to a quality check and then analyzed using the theme analysis method developed by Braun and Clarke (46). It followed coding steps and necessitated the transcription of interview tapes. In the first step, writers reviewed and reread transcripts to pick up prospective topics, which they then sent to the head writer. The second analysis phase involved the original and final writers looking at these preliminary codes. They seriously considered how to create overarching motifs and higher-level subthemes while preserving the original codes' variety. Third, the quotations consistent with the overall themes were discovered via analysis undertaken by the first and final writers. After that, the writers looked through topics before defining and naming them. Once the report's concepts were finalized–by both the first and last authors–the actual writing could begin.

## Results

### Derived themes and sub-themes

#### E-social prescription

**Figure 2:**
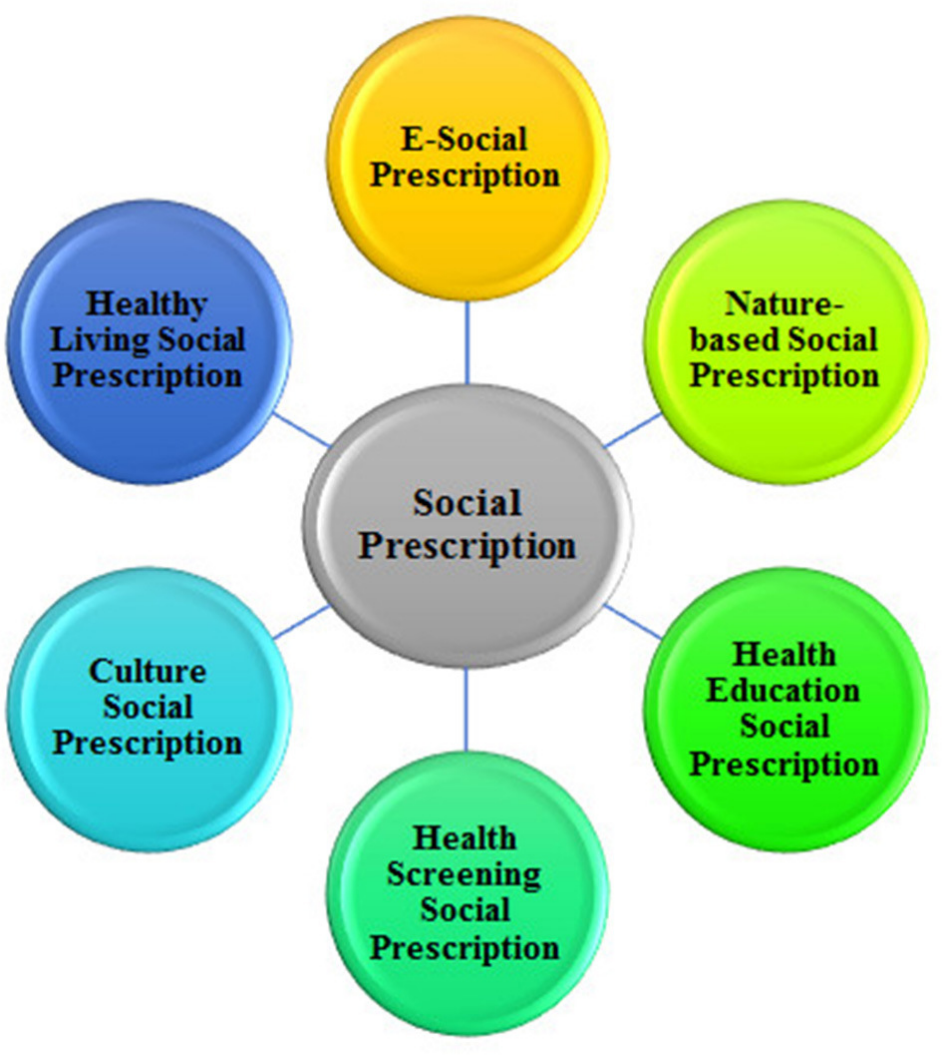
Main themes.

The theme of E-Social Prescription revolves around utilizing technology to enhance social connectedness, wellbeing, and overall health through various activities. All the study participants know the importance of “E-Social Prescription” and recommend it to the Chinese older adult for healthy aging (see Table 2 and Figure 2).

**Table 2:** Derived themes and sub-themes.

**Main theme**	**Sub-theme**	**Context/Activities**
E-social prescription	Smart mobile phone groups for senior citizen	Telecare services, Digital literacy, Wechat, and Q.Q. Groups for social connectedness, E-learning, Wearable fitness, and health trackers (Health monitoring and management), Online healthcare community
Nature-based social prescription	Activities in the green community gardens, walk-in green and wetland parks	Tai Chi and Qigong in the green community and public parks, Outdoor walks in the green and wetland parks, gardening, vegetation, and others
Healthy living social prescription	Behavioral health	Nutritious meals and dietary counseling, Smoking cessation, reducing alcohol consumption, physical activity
Culture social prescription	Culture and Social Engagement	Group outings, card games, board games, singing, dancing, arts and crafts activities, love confession activity
Health screening social prescription	Preventive health	Blood sugar check-up, Blood pressure check-up, Ophthalmic, dental, and physical examination
Health education social prescription	Health awareness	Lectures on health management, peer education regarding health, distribution of brochures, flyers, and posters containing health awareness information

*E-Social prescription aims to enhance individuals' overall health and wellbeing by fostering social support, promoting health monitoring and management, and providing continuous learning and engagement opportunities. (CETM 1, 3)*

*An e-Social prescription is a multifaceted approach that leverages digital tools such as telecare services, social media platforms, wearable fitness and health trackers, e-learning, and online healthcare communities to promote social connectedness, digital literacy, and health monitoring and management, thereby enhancing the overall wellbeing of individuals and communities. (CETM, 5, 7)*

*Smart mobile phones can provide a range of services to senior citizens, including telecare services, digital literacy training, social connection, knowledge enhancement, health monitoring and management, and support and information sharing. (CETM, 22, 24)*

*Smart mobile phone groups can provide several benefits to senior citizens. These groups can improve health and wellbeing, increase social connection, enhance cognitive function, reduce isolation, and increase independence. (CETM, 2, 17)*

### Nature-based social prescription

The theme of Nature-based Social Prescription emphasizes the holistic wellbeing of individuals through engaging in nature-based activities. It recognizes the intrinsic connection between nature and human health, promoting physical activity, relaxation, social interaction, and a sense of purpose within natural environments, thus enhancing overall health and quality of life. All study participants know the importance of “Nature-based Social Prescription” and acknowledge its role in promoting healthy aging (see Table 2 and Figure 2).

*Nature-based social prescription activities, such as Tai Chi & Qigong in green community gardens, outdoor walking in green parks, and gardening, provide opportunities for individuals to connect with nature, enhance their physical and mental wellbeing, and promote social interactions and community engagement. These activities also offer a sustainable and cost-effective approach to improving health outcomes and promoting environmental awareness and conservation. (CETM, 21, 4)*

*Nature-based social prescription (NBSP) is an intervention that uses exposure to nature to improve health and wellbeing. NBSP interventions can be delivered in various settings, including green community gardens, green parks, and other natural areas. (CETM, 11, 3)*

*NBSP is a promising intervention that can be used to improve health and wellbeing. NBSP interventions can be delivered in various settings and tailored to the individual's needs and interests. (CETM, 19, 6)*

### Healthy living social prescription

This thematic statement captures the essence of healthy living initiatives as social prescriptions. It highlights the healthy living initiatives' comprehensive nature and potential impact on individual wellbeing, emphasizing the holistic approach to improving health through social interventions. Healthy living initiatives as social prescription offer a holistic approach to healthcare, addressing physical health and promoting mental and emotional wellbeing (see Table 2 and Figure 2).

*Through activities such as nutritious meals and dietary counseling, smoking cessation, reducing alcohol consumption, and physical activity, individuals can improve their overall health, increase their self-esteem, and foster a sense of community and social support. Such initiatives prevent chronic diseases, reduce healthcare costs, and create a healthier society. (CETM, 11, 3)*

*Healthy living initiatives as the social prescription is a way to improve the health and wellbeing of individuals and communities by providing them with access to resources and support that can help them make healthy lifestyle choices. (CETM, 17, 2)*

### Culture-based social prescription

The theme of Culture Social Prescription explores the therapeutic benefits of diverse cultural activities, promoting social connection, self-expression, and emotional wellbeing. Cultural activities allow individuals to connect with others, express themselves creatively, and explore different cultural experiences, ultimately leading to a sense of belonging and improved overall quality of life (see Table 2 and Figure 2).

*Through group outings, card games, singing, dancing, arts and crafts activities, love confession activities, and opera, individuals engage in culturally rich experiences that foster personal growth, community bonds, and holistic healing. (CETM, 5, 7)*

*Cultural, social prescriptions, such as group outings, card games, singing, dancing, arts and crafts activities, love confession activities, and opera, provide individuals with opportunities to engage in meaningful and enjoyable social interactions that promote mental and emotional wellbeing. (CETM, 11, 13)*

*Culture-social prescription is an intervention that uses cultural activities to improve health and wellbeing. It can be delivered in various settings, including community centers, libraries, and museums. (CETM, 21, 4)*

*By providing people access to cultural activities, we can help them improve their health and wellbeing and reduce the burden of chronic disease on our communities. (CETM, 19, 6)*

### Health screening social prescription

The Health screening Social Prescription theme encompasses a comprehensive approach to preventive healthcare. It emphasizes the importance of early detection, monitoring, and intervention to promote overall wellbeing and prevent the progression of health conditions conditions (see Table 2 and Figure 2).

*By integrating these screening activities into social prescriptions, individuals are empowered to take proactive measures for their health, leading to improved healthcare outcomes and enhanced quality of life. (CETM, 1, 3: CETM 11, 13)*

*By implementing health screening social prescription, individuals are empowered to proactively monitor their health through regular blood sugar and blood pressure check-up and ophthalmic, dental, and physical examinations, promoting early detection and prevention of potential health issues. (CETM, 17, 2: CETM, 22, 24)*

*Health screening social prescription is an intervention that uses health screenings to improve health and wellbeing. Health screening social prescriptions can be delivered in various settings, including community health centers, hospitals, and doctor's offices. (CETM, 19, 6)*

### Health education social prescription

Health education as social prescription is an intervention that uses health education to improve health and wellbeing. It can be delivered in various settings, including community health centers (see Table 2 and Figure 2).

*Implementing diverse and accessible health education strategies, such as lectures on health management, peer education, and distributing brochures, flyers, and posters to Chinese older adult, promotes holistic wellbeing and empowers individuals to actively engage in self-care and disease prevention. (CETM, 11, 13: CETM, 21, 4)*

*The theme of “Health Education Social Prescription” aims to promote healthy living and wellbeing through various educational activities such as lectures on health management, peer education, and distributing brochures, flyers, and posters to the Chinese older adult. These activities serve as valuable resources for disseminating important health information and encouraging behavioral changes that can lead to improved health outcomes and a better quality of life for older adults in the Chinese community. (CETM, 19, 6: CETM, 5, 7)*

## Discussion

In the Zhejiang Province of China, social care techniques for senior citizens often comprise linking people to neighborhood volunteer and community services, as well as giving access to psychotherapy and lifestyle guidance. In addition, older adult care procedures can aid in addressing psychological and social problems that may be a factor in a person's illness (51). In the context of the social prescription, we analyzed social healthcare practices for healthy aging at the community level. All the comprehensive evaluation team members described community social healthcare practices under the paradigm of social prescription. The three primary facets of community care are daycare services, in-home caregiving assistance, and participation in leisure activities like sports and cultural events. The central government releases a variety of policy initiatives to entice and encourage service providers to engage in the senior care field to build capability (52). To evaluate the efficacy of various initiatives and support services for senior citizens in Zhejiang Province, the government has started trial assessment mechanisms in a few areas. Older adults can also benefit from community health support programs that include health promotion, coaching, and health education (51). Plans for older adult care policy ask local governments to develop senior education and recreation facilities to support healthy aging (53). China has been advancing older people's access to integrated care through a community-based social and health care system. Integrated community-based social and health care system includes prevention and control of chronic disease, increased health services, and a trained health workforce (54).

In a community, various non-medical services are offered, such as social security benefits, volunteer opportunities, fellowship gatherings, training possibilities, and chances to exercise and study (55). Wellbeing results from numerous variables, aside from medical, behavioral, and social facets of life, which also present the potential for healthcare. Those with protracted or chronic mental and physical disorders must make a special effort to adopt healthy behaviors. According to definitions, social prescribing is a “clear, coherent, and collaborative procedure in which healthcare professionals collaborate with patients and service users to choose and refer to the available services in the community (51).” A “social prescribing” initiative was implemented by the National Health Service (NHS) of the U.K. in 2016 to combat depression and loneliness among U.K. citizens while easing the workload for general practitioners (GPs). Connecting individuals with non-medical community services is referred to as “social prescribing,” which provides a service that extends beyond medical therapy for a sickness (56). The concept of “social prescribing” came into existence due to the significant contribution that the community may make to overall wellness and health. Social prescribing directs People to several non-clinical and non-health resources (57). Social prescribing uses a patient- and values-centered psychological method to assist people in recognizing the adverse social drivers of the present situation, address them, and devise a joint strategy to remedy them. SP is defined as “a pathway to refer clients to non-clinical services; linking clients to community support to improve overall health, to foster life quality, to promote consciousness wherever suitable, and to reduce vulnerability inside the society alongside the person (58).”

Using computers and mobile applications may also assist individuals in leading healthier lives and experiencing fewer health issues as they age (59). The government supports smart digital technology because it understands how technical advancement may make home care easier (53). Older people use robotics, monitoring, call-service platforms, and virtual nursing systems (53). Older adult Chinese citizens like to get their care at home, but when assistance is required, they can also use community services. Healthcare has risen in importance in policy over the last 10 years, and the Chinese government is putting a lot of effort into creating smart home care technologies (54). Social media apps use the Internet and Web 2.0 technology to let users engage in different virtual communities and produce and exchange information through features like communicating, exchanging, working together, posting, and connecting (60). According to the findings of our study, smart mobile phone groups (Wechat and Q.Q. groups) for senior Chinese citizens were created at the community level. The purpose of these groups is telecare services, digital literacy, social connectedness, e-learning, guidance about wearable fitness and health trackers (Health monitoring and management), and online healthcare services. All these activities come under “E-social Prescription” and are vital in promoting healthy aging at the community level. Call-service platforms and virtual nursing systems are encouraged to be established by the 12th and 13th FYPs. Automation, artificial intelligence, cloud technology, and the new generation of digital technologies are advancing quickly. The Guideline Views of Actively Encouraging Internet + Activities were released by the central government in 2015, and in 2017 they were followed by “The Action Plan for the Growth of Smart Health and the Aged Care Industry (2017–2020)”. To enter the Internet - of - things sector emphasizing community and home care activities (61). Older adult care services have developed into the fourth scientific and technological revolution, defined by the Internet of Things (IoTs), information technology, big data, and cloud services. The development of aging-in-place has been facilitated by several emerging technologies, one of which is smart home technology for older people to address their senior's needs, including comfort, freedom, healthcare, and help. Smart Home for senior care is committed to offering various reasonably priced services (62, 63). In China, utilizing the Wechat app for communication is prevalent. The “hypertension management group” comprised patients who used the Wechat mobile app. Therefore, the “kangkang shengshi” public account was created. In this Wechat community, general practitioners frequently provide information about developing and preventing hypertension through daily posts. Members of the Social messaging group were urged to interact with each other, express their emotions, and support each other (64).

Older people are invited to participate in social functions, volunteer, and participate in economic pursuits, such as starting their enterprises (13th FYP). It is frequently stressed how important physical activity is for older adults. Older adults should exercise more than 50% of the time (12th FYP) (55). Our findings indicated that CETMs recommend nature and culture-based activities for healthy aging at the community level to foster social connectedness. Using nature as therapy is a subset of the larger social prescribing trend, which promotes utilizing additional actions as efficient and frequently low-cost therapies, such as exercise, socialization, house enhancements, and other things (65). The benefits of living in a neighborhood that encourages encounters with water or “blue spaces” may also extend to mental wellness. Better mental health is linked to living near nature, participating in outdoor activities, and experiencing a connection (66). Our findings show that CETM's recommended nature-based activities such as Tai Chi and Qigong in the green community and public parks, Outdoor walks in the green and wetland parks, gardening, and vegetation. Group outings, card games, board games, singing, dancing, arts and crafts activities, and love confession activity for social engagement under culture prescription. Blue space is “all visible, outdoor, natural surface waters to promote human health and wellness.” Stream, river, and coastal regions are examples of blue space (67). A social prescription is a recommendation that enables you to interact with your community; it's not a medication that comes in a pharmacy bottle or blister pack. Under social prescription, participation in communal activities such as the arts, education, athletics, reading, or volunteering is advised, as well as seeking guidance and assistance regarding one's health and wellbeing (68). Medical practitioners and policymakers are increasingly promoting using nature-based health interventions (NBIs) and conventional medication and psychological therapies to meet the rising demands associated with poor mental health. A growing amount of research indicates that nature can help those with mental disorders by reducing their suffering. Along a wide range of mental health indicators, blue spaces, defined as environments mainly consisting of water, have been demonstrated to be more effective at boosting wellbeing than green spaces (69, 70).

Social prescribing has become an effective tool for assisting patients in overcoming some of the social and behavioral causes of poor wellbeing (71). All the study participants reported that health behavior and health awareness under social prescription are vital for healthy aging among Chinese seniors. Doctors, sociologists, associated medical practitioners, members of the charity sector, and those involved in commissions have all shown an increased devotion to social prescribing. It has aided physicians in convincing appropriate patients to engage in novel and beneficial behavior changes. It uses a patient- and values-centered psychological method to assist people in recognizing the adverse social drivers of the present situation, address them, and devise a joint strategy to remedy them. SP is “a pathway to refer clients to non-clinical services; linking clients to community support to improve overall health, to foster life quality, to promote consciousness wherever suitable, and to reduce vulnerability inside the society alongside the person (72).” Individuals in China also benefit from music therapy and dance therapy to promote good aging. There is a common misunderstanding that these treatments improve mental health and brain function (5). Giving older adult individuals emotional support and entertainment in the neighborhood helps lessen psychological issues like sadness and anxiety and further safeguards their brain health (73). Daily living assistance, medical assistance, and health services frequently impact older people's ability to think clearly. Adverse affective feelings, including despair and anxiousness, have been linked to cognitive decline (74). Care in the community has become a way for older adults to receive daycare while still living at home, either through visiting services or by residing in a daycare facility. Older persons who follow this plan could occasionally receive assistance from family members rather than having to leave the house (75).

Art-based therapies in China have improved patients' mental and emotional wellbeing. A patient with schizophrenia benefit from group art therapy incorporating traditional Chinese objects because it increases their sense of competence and social functioning, decrease their difficulties with these areas of functioning, and enhances their overall quality of life (76). Promoting older individuals' health can be aided through social engagement, a crucial active living component. Older people should have a better quality of life to maintain their wellbeing, social contact, and optimal health. Social engagement is one of the elements of active aging. It is a positive activity where individuals and groups actively and willingly participate in community events to connect and communicate with one another (77). Healthcare consciousness is one of the crucial ways to enhance the health status of older persons, who typically suffer from several long-term illnesses. Older adults who live in the community, as opposed to those who are institutionalized, are more independent, require more health information, and want to maximize their opportunities for participation, health, and security in to enhance their standard of living, a direct indication of successful aging (78). All the study participants reported about health screening activities for older adults at the community level. Health screening is a new component of the traditional care system for healthy aging. Integrating social and medical care for older people in China is a relatively new concept. The State Council has been releasing “Many Views on Accelerated the Growth of the Older adult Care Sector” since 2013, suggesting expanding traditional older adult care to include medical treatment and create a thriving old-age care service sector known as “Yiyang Jiehe” in Chinese (79).

## Conclusion

Prescription of non-medical interventions can be considered an example of social prescription, which can potentially promote health and wellbeing on a community level in China. In China, significant components of social medicine include encouraging people to engage in physical activity, assisting individuals in developing their social capital, and enhancing the quality of life in their communities. Health professionals often refer people to community social events to improve their health and wellbeing. At the community level in China, “social prescription” refers to the practice in which healthcare practitioners advise their patients to engage in activities that are not medical to improve their health and overall quality of life. Among these interventions are programs for physical activity, events for social interaction, and activities in natural settings. At this point, the moral imperative of social prescribing makes it abundantly evident that action must be taken concerning this topic. As part of a social prescription for good aging, non-medical interventions are utilized to improve the health and wellbeing of older adults. It aims to enhance the quality of life for the older adult population. The social prescription for healthy aging includes active participation in one's local community. It also demonstrates that health and social care employees have a new responsibility to act as a bridge between public services and local communities to assist individuals struggling with various health issues in becoming active members of their communities. The policy of social prescribing advocates for a gradual implementation that will be concentrated on primary care network organizations.

## Implications

The outcomes of the investigation carry several implications.

### Theoretical implications

The notion of “social prescription” is congruent with the fundamental tenets of social medicine. The proposition posits that healthcare practitioners in China ought to broaden their scope beyond medical therapies and incorporate non-medical approaches to augment the holistic wellbeing of individuals and communities. The research emphasizes the significance of implementing interventions at the community level to enhance health and wellbeing. It highlights that strengthening social capital, participating in physical activities, and nurturing community quality of life can be as crucial to medical therapies. Implementing social prescribing requires a collaborative effort between healthcare professionals and community organizations, emphasizing the importance of multidisciplinary collaboration. The study suggests a greater emphasis on interdisciplinary collaboration is necessary to effectively connect healthcare services with community resources.

### The social and applied implications

These implications encompass the impact on individuals, communities, and society as a whole. Developing and implementing social prescribing programmes should involve collaboration between health organizations and community groups. It is recommended that these programmes provide a diverse range of non-medical interventions, including physical exercise programmes and social events, to those who are actively striving to enhance their overall wellbeing. Healthcare professionals must undergo comprehensive training to acquire the necessary skills to proficiently prescribe non-medical therapies and facilitate the proper linkage of patients with suitable community resources. Integrating this training into medical education and continuous professional growth is crucial. Integrating social prescribing within primary care network organizations can be a viable paradigm for gradually deploying these practices within the healthcare system.

### The policy implications

The study's outcomes underscore the necessity of legislative modifications that facilitate the integration of social prescription into the healthcare system. This necessitates a transition toward a progressive amalgamation, specifically emphasizing organizations dedicated to primary care networks. Policymakers should deliberate the formulation of guidelines, implementation of training programmes, and establishment of support structures to facilitate the proficient prescription and supervision of non-medical interventions by healthcare professionals. Furthermore, the allocation of funds and distribution of resources must align with the recognition of social prescription as a legitimate healthcare strategy.

### Future scope of the study

The study's conclusion suggests an opportunity for a more comprehensive healthcare approach in China, emphasizing the potential of social prescription to enhance community health and wellbeing. This statement suggests that additional research and policy development are necessary to effectively utilize the advantages of this strategy and establish a healthcare system that is comprehensive and interconnected. This work offers significant contributions to understanding social prescription in the context of China. However, it also highlights various potential areas for future research.

#### Evaluation of effectiveness

Undertake comprehensive research to evaluate the efficacy of many non-medical interventions recommended via social prescribing. It will facilitate a more extensive comprehension of the activities that produce the most substantial advantages for health and wellbeing.

#### Long-term health outcomes

Investigating the enduring health consequences associated with the implementation of non-medical interventions. It may entail conducting longitudinal research to ascertain whether continuous engagement in activity diminishes healthcare burdens and enhances overall health outcomes in individuals.

#### Technology integration in social prescription

Technology integration has become increasingly prevalent in the healthcare field, particularly in the realm of digital health solutions. As such, future research endeavors can focus on exploring the potential benefits of technology in improving the delivery and accessibility of social prescribing interventions.

### Study limitations

The research possesses certain constraints that necessitate careful consideration when evaluating its results. The study utilized a purposive sampling technique to recruit participants from the “comprehensive evaluation team,” which may have introduced bias in the sample selection process. The presence of this bias can potentially restrict the results' applicability to a broader range of healthcare professionals or those with a vested interest in the field. Moreover, the research's emphasis on Yiwu, located in Zhejiang Province, China, may lead to a restricted geographical perspective, thus reducing the generalizability of the results to other areas or nations characterized by unique healthcare environments.

## Data availability statement

The datasets for this manuscript are not publicly available due to ethical requirements. Requests to access the datasets should be directed to the corresponding author(s).

## Ethics statement

The studies involving humans were approved by the Fourth Affiliated Hospital of Zhejiang University, School of Medicine, Human Research Ethics Committee approval no: K2023034. The studies were conducted in accordance with the local legislation and institutional requirements. The participants provided their written informed consent to participate in this study.

## Author contributions

RM and LY: methodology and conceptualization. RM and RDN: validation, software, formal analysis, and original draft writing. LY: supervision, reviewing, and editing. RM: wrote the final article. All authors contributed to the article and approved the submitted version.
